# Duration of Immunity after Vaccination

**Published:** 1898-09

**Authors:** 


### DURATION OF IMMUNITY AFTER VACCINATION.

According to Jasiewicz, the immunity from vaccination lasts a much shorter time in infants than is generally supposed. A study of the statistics of the subject showed a proportion of 7.35 per cent. out of twenty-three children under six years of age in whom vaccination was successfully performed. The author, therefore, recommends more frequent revaccination in childhood and expresses the opinion that children are also protected from other infectious diseases thereby.—*Jour. de Clin. et Ther de l'Enfants.—Universal Medical Journal.*

